# BST2 induced macrophage M2 polarization to promote the progression of colorectal cancer

**DOI:** 10.7150/ijbs.72538

**Published:** 2023-01-01

**Authors:** Xuefeng He, Huaijun Chen, Xinyang Zhong, Yaxian Wang, Zijuan Hu, Huixia Huang, Senlin Zhao, Ping Wei, Debing Shi, Dawei Li

**Affiliations:** 1 Department of Colorectal Surgery, Fudan University Shanghai Cancer Center, Shanghai, China; 2 Department of Pathology, Fudan University Shanghai Cancer Center, Shanghai, China; 3 Cancer Institute, Fudan University Shanghai Cancer Center, Shanghai, China; 4 Institute of Pathology, Fudan University, Shanghai, China; 5 Department of Oncology, Shanghai Medical College Fudan University, Shanghai, China; 6 Department of Neurosurgery, The Second Affiliated Hospital of Zhejiang University School of Medicine, Hangzhou, China

**Keywords:** BST2, Colorectal cancer (CRC), M2 macrophages, Biomarker

## Abstract

**Background:** Tumor-associated macrophages (TAMs) are one of the most prominent tumor-infiltrating immune cells in the tumor microenvironment (TME) of CRC and play a vital role in the progression of CRC. BST2 was predicted to be associated with the infiltration of TAMs. However, its potential function by which CRC cells and TAMs interact with each other still needs further investigation.

**Methods:** The target genes in CRC were selected by bioinformatics screening. The level of bone marrow stromal cell antigen 2 (BST2) in CRC cells and tissues was determined by qRT‒PCR, Western blotting, and immunohistochemistry staining. *In vitro* and *in vivo* assays were applied to clarify the function of BST2.

**Results:** In this study, according to bioinformatics analysis, a nomogram based on the risk score (constructed by BST2 and CAV1 (caveolin-1)) and clinical features was built and displayed satisfactory prognostic value. Upregulated BST2 was significantly related to Braf mutation, dMMR/MSI-H, CMS1 subtype, and immune response and was a potential biomarker for predicting immune checkpoint blockade therapy. Silencing BST2 in CRC obviously restrained CRC progression and M2 TAM polarization. The infiltration of TAMs was positively correlated with the high expression of BST2, and depletion of TAMs alleviated the protumoural effect of BST2 in CRC *in vivo*. *In vitro* experiments revealed that a reduction in BST2 in CRC inhibited CRC proliferation and migration and also M2 polarization.

**Conclusion:** These findings indicated that BST2 played a vital role in CRC progression and might be a predictable marker for immunotherapy.

## Introduction

Colorectal cancer (CRC) is one of the most commonly diagnosed cancers worldwide, and the death rate ranks second, with 10% of tumor-related deaths annually 1. With the improvement of biotechnologies and therapeutic strategies, such as combination systemic therapies and immunotherapy, the past two decades have witnessed a gradual decline in the incidence and disease-specific mortality 2, 3. However, the five-year survival of metastatic colorectal cancer patients still remains poor at only approximately 14% 4. The tumor microenvironment (TME) is considered to be of great importance in CRC progression 5. Thus, determining how the components of the TME interact with CRC cells and finding a better therapeutic strategy for CRC patients would be helpful.

Tumor-associated macrophages (TAMs) are deemed to be one of the most prominent tumor-infiltrating immune cells in the crosstalk between cancer cells and the TME 6-8. Notably, macrophages experience dynamic alterations in phenotypes and respond to diverse protumoral activities. Moreover, different tumors possess a various spectrum of TAMs mainly influenced by the specific tumor microenvironment 9-11. Accumulating evidence has demonstrated that TAMs promote the proliferation, migration, invasion, metastasis and chemoresistance of CRC. A high density of infiltrating TAMs is often positively correlated with poor prognosis in CRC 10, 12. M2-TAMs tend to localize in the most active areas of tumors to promote the progression of tumors; typically, CRC with advanced stages has a high tendency to be infiltrated with M2 phenotypes 10, 13, 14. Nevertheless, the detailed interaction between CRC cells and TAMs still remains to be further explored.

Based on bioinformatic analysis and document retrieval, BST2 (bone marrow stromal cell antigen 2, also named HM1.24/CD317) was selected for further study. A high level of BST2 was significantly correlated with Braf mutation, dMMR/MSI-H and immune response; and was a potential biomarker for predicting immunotherapy response. The integration of our results revealed that BST2 was crucial for the communication between CRC cells and M2 TAMs. Known as a type II transmembrane protein, BST2 has been validated to play an oncogenic role in myeloma, breast cancer, lung cancer and kidney cancers 15-18. Previous studies indicated that immunotherapy with a monoclonal antibody against BST2 could not only trigger antibody-dependent cellular cytotoxicity and complement-dependent cytotoxicity but also reduce tumor size and extend survival to some extent in myeloma 19. A small number of studies focusing on CRC have revealed BST2 could be an independent prognostic biomarker 20, 21. However, the deeper investigation of how BST2 might affect the development of CRC is still largely unknown. Our present study revealed the crucial role of BST2 in facilitating cell growth and migration and provided evidence for the correlation between CRC cells and TAMs.

## Materials and methods

### The acquisition of data and the calculation of estimate scores

The RNA-seq dataset of TCGA-COAD and clinical data of CRC patients were downloaded from https://xenabrowser.net/. Gene expression data of GSE39582, GSE33113, GSE38832, GSE17536 and GSE179351 can be found on the website https://www.ncbi.nlm.nih.gov/geo/, which is the GEO database. The calculation of immune and stromal scores was analyzed by the ESTIMATE algorithm via the R package “estimate” 22.

### DEG analysis and WGCNA analysis

Differentially expressed gene (DEG) analysis was conducted by adopting the edgeR R package. The definition of DEGs was genes with false discovery rate (FDR) value < 0.05 and |Log2 (fold change (FC))| > 1. Coexpressed gene modules were constructed by weighted correlation network analysis (WGCNA). These modules were recognized to be closely relevant to immune and stromal scores. The positive TME-correlated modules had a correlation coefficient > 0.5.

### TME-related risk score development

The genes related to immune and stromal factors that were determined by the intersection of DEGs and WGCNA were subsequently inputted into the LASSO (Least absolute shrinkage and selection operator) regression analysis and subsequent univariate Cox regression analyses. When faced with a univariate Cox P value <0.05, these genes were subjected to stepwise Cox regression based on the AIC. The risk score was established using the following formula: risk score= β1×the expression of Gene 1+…+ βn × the expression of Gene n. In the model, β represented the quotient of each gene with the minimal AIC value.

### Building and validating of the nomogram

The prognostic nomogram was constructed by the integration of risk score, TNM stage, age and sex to predict the overall survival probability at 1, 3, and 5 years for CRC patients. It was constructed by using the rms R package. The concordance index (C‐index) was calculated to estimate the discrimination of the nomogram, while the calibration curves were utilized to assess the association between the predicted and observed risk for the outcomes of the nomogram.

### Immune signature and GSEA for BST2 in the TCGA database

The correlation between HLA family genes/immune checkpoints and BST2 expression was demonstrated by correlation analysis in the TCGA-COAD database. TIMER, CIBERSORT, and xCell algorithms were used to determine the potential infiltrating immune and stromal cell types, which are available in the TIMER 2.0 database (http://timer.comp-genomics.org/). The possible biological functions of BST2 were analyzed by using the GSEA tool in the TCGA-COAD dataset. The Gene Ontology (GO) and Kyoto Encyclopedia of Genes and Genomes (KEGG) databases were used to annotate biological pathways by utilizing the clusterProfiler R package.

### Single-cell RNA-seq analysis

The processed Smart-seq2 gene expression data and metadata were obtained from GSE146771. GSEA of monocytes/macrophages (including monocytes, macrophages and TAMs) and malignant cells was performed as described above. The crosstalk between monocytes/macrophages and malignant cells was identified by the iTALK R package.

### Cultivation of human and mouse CRC cell lines

The human CRC cell line RKO and the murine cell line MC-38 were acquired from ATCC and cultured in 5% CO_2_ at 37 ℃ in DMEM (Gibco) supplemented with glutamine (2 mM), nonessential amino acids (0.1 mM), sodium pyruvate (1 mM), 1% penicillin/streptomycin and 10% fetal bovine serum (FBS). The human monocytic cell line THP-1 was also acquired from ATCC and was cultured with RPMI 1640 medium (Gibco, USA) that contained 1% penicillin/streptomycin (Gibco, USA) and 10% fetal bovine serum (Invitrogen, USA).

### Coculture of induced macrophages and CRC cells

THP-1 cells (1×10^6^) were seeded in 6 well plate, and then phorbol 12-myristate 13-acetate (PMA; 100 ng/mL; Shanghai Maokang Biotechnology, China) was applied to induce the differentiation of THP-1 cells into macrophages for approximately 12 hours in 5% CO_2_ at 37 ℃. After 12 hours, the cells would largely adhere to the wall. Replace the medium after 12 hours and add IL-4 (20 ng/mL) and IL-13 (20 ng/mL) to induce M2 macrophage polarization for approximately 24 hours. After induction, the chambers (corning, 24 mm diameter, 0.4 µm polycarbonate membrane, #3412) loaded with BST2 knockdown CRC cells (2 × 10^5^) were put on the upper level of the wells, and the CRC cells were cocultured with macrophages for another 24 hours. After the whole process, either CRC cells or macrophages could be analyzed with different assays.

### Transient transfection and quantitative real-time PCR (qRT‒PCR)

RNA extraction was extracted by NucleoZOL reagent (Macherey-Nagel, Germany). Reverse transcription was performed by ABScript II RT Mix for qPCR (ABclonal, China). qRT‒PCR was performed with TB Green® Premix Ex Taq™ II (TaKaRa, Japan). Negative control (NC) and BST2 siRNA (GenePharma, Shanghai, China) were transfected into the RKO cell with the guidance of the manufacturer's instruction of Lipofectamine RNAiMAX (Invitrogen). The target sequences for BST2 siRNA were GCUCCUGAUCAUCGUGAUUTT and GCAAUGUCACCCAUCUCCUTT. The qRT‒PCR primer sequences were listed as follows: hβ-Actin: forward primer-5′-ACCGAGCGCGGCTACAG-3′, reverse primer-5′-CTTAATGTCACGCACGATTTCC-3′; hBST2: forward primer-5′-CACACTGTGATGGCCCTAATG-3′; reverse primer-5′-GTCCGCGATTCTCACGCTT -3′.

### Western blotting

The proteins were acquired from cell lines using RIPA buffer (Thermo) containing a protease inhibitor (Bimake), and then immunoblotting was performed. A bicinchoninic acid protein assay kit was applied to quantify the protein content of the cell lysates. Protein samples were loaded into PAGE gels (Epizyme Biomedical Technology, Shanghai, China) and transferred to 0.2 µm immobilon PVDF membranes (Millipore Sigma). After blocking with quick blocking buffer (QuickBlock™, Beyotime ), membranes were covered with primary antibodies in proper dilutions overnight at 4 °C and secondary antibodies were incubated at room temperature for approximately 1 hour and then visualized by an ECL system (Share-bio, Shanghai, China). The dilution factor of primary antibodies against BST2 (ab88523) and PD-L1(ab213480) was 1:1000, and GAPDH (Proteintech, 60004-1-Ig) was 1:5000. Secondary antibodies included anti-mouse IgG (Proteintech, SA00001-1) and anti-rabbit IgG (Proteintech, SA00001-2 ), and all the secondary antibodies were diluted at 1:5000.

### Immunohistochemistry (IHC) staining

All the tissue samples for IHC staining were approved by the Ethics Committee of the Fudan University Shanghai Cancer Center (Shanghai, China). Briefly, paraffin-embedded tissue slices were deparaffinized in three different tanks of xylene and rehydrated in a graded ethanol tank by tank. Antigen retrieval was conducted at pH 8.0 and 120 °C at full pressure, made by autoclaving for approximately 10 minutes, followed by inactivation of endogenous peroxidase activity with 3% H_2_O_2_ diluted in methanol. Following nonspecific binding, antibody incubation for tissue slices was prepared at 4 °C for 12 hours, and the primary antibodies were BST2 (ab88523, 1:200 dilution), CD163(ab182422, 1:200 dilution), CD3 (ab5690, 1:200 dilution) and CD33 (ab269456, 1:200 dilution). Secondary antibody (GK600510, Genetech, Shanghai, China; 50-100 µL each) was incubated at room temperature for approximately 45 minutes. The staining time for 3,3 -diaminobenzidine (DAB) was tissue dependent, and that for Mayer's hematoxylin solution was 5-10 minutes. After acquiring images, the mean value calculated for positive immune cells from five random fields at 20× magnification was used for downstream analysis. Scoring was conducted according to the immunoreactive score (IRS) standard as previously reported 23, and the IRS value higher than the mean value was included in the high-expression group (23 samples) and lower than the mean value was included in the low-expression group (17 samples).

### Cell counting kit-8 proliferation assay (CCK-8)

Briefly, approximately 1.5 × 10^3^ RKO cells were seeded into 96-well plates per well with 5 repetitions in each group. After the cells adhered, 10 μL of CCK-8 reagent (Yeasen) was added to 100 uL of cell culture medium and mixed thoroughly; then, the mixed reagent was added to each well of the whole plate. After that, the 96-well plate was and incubated at 37 °C for 2 hours in the dark. The whole plate was then taken into the Biotek system to measure the absorbance value at 450 nm. The cell proliferation curves were plotted as the measured values of each day by deleting the minimum and maximum values.

### Transwell Assay

RKO cells (6 × 10^4^) were seeded in the upper chamber of a Transwell filter with 8-μm pores (#3422, Corning) with serum-free medium, and the chamber was loaded with total medium containing 10% FBS. After 48 hours, the cells would migrate to the underside of the chamber, and the cells were fixed with 4% paraformaldehyde (Beyotime) and then stained with 0.4% crystal violet (Yeasen Biotechnology). The nonmigrated cells in the upper chamber were completely removed by cotton swabs and washed with PBS. The relative cell migration was compared between the treated group and the control group. The number of cells was counted from five random fields at 100× magnification on the underside of the chamber.

### Animal experiments

Wild-type female C57BL/6 mice (6-7 weeks old) were purchased from the Cavens Laboratory and housed in specific pathogen-free conditions. Each cage for each group was housed with no more than 6 mice. All experiments were performed in accordance with the animal care guidelines of the Institutional Animal Care and Use Committee (IACUC) of Fudan University Shanghai Medical School. The mice were randomly divided into treated groups and a control group according to the experimental needs. MC38 cells (1 × 10^6^) were subcutaneously injected into the right flank of the C57BL/6 mice in each group, and for the macrophage-depletion model, C57BL/6 mice were intraperitoneally injected with 150 µL clodronate liposomes or control liposomes (FormuMax Scientific, USA) before cancer cell injection. The measurement of tumor volumes was performed every two days by recording the length (x) and width (y) of each model, and the calculation forum for tumor volume was 0.5 × (xy^2^). After the difference gradually appeared between different groups, mice were euthanized, and tumors were dissected for further study. The average volume should not be more than 1500 mm^3^ in order to alleviate the suffering of the mice.

### Flow cytometry analysis

The dissociated tumors were dissociated into single-cell suspensions according to the Tumor Dissociation Kit (Miltenyi Biotec), followed by filtering dissociated cells through a 70-µm nylon mesh filter (WHB-70 µm, WHB Scientific, Shanghai, China). After the blocking with TruStain FcX™ (anti-mouse CD16/32) (Biolegend, 101320), the cells were incubated for 30 minutes with primary antibodies purchased from BioLegend including mouse CD45 (1:100 dilution), mouse F4/80 (1:100 dilution) and mouse CD11b (1:100 dilition).

Macrophages induced from THP-1 cells were harvested and collected using cold PBS. The cells were resuspended in flow cytometry buffer (1× PBS buffer containing 1% BSA), stained with anti-CD163 antibody (BioLegend, #333618, 1:100 dilution) and anti-CD206 antibody (BioLegend, #321110, 1:100 dilution) for 30 minutes on ice and then analyzed using flow cytometry (C500, Beckman, USA). The data was analyzed using FlowJo software.

### Cell cycle

The treated RKO cells were harvested and collected in centrifuge tubes. Then, cold 75% ethanol was added to tubes to fix the cells at -20 °C for approximately 12 hours. Subsequently, we used PBS to wash samples three times. Afterward, propidium iodide (PI) (BD Biosciences, USA) was applied to stain the samples for 30 minutes at room temperature in the dark. After all the preparations, flow cytometry (FC500, Beckman, USA) was used to analyze the cell cycle according to the manufacturer's protocol.

### Statistical analysis

For normally distributed variables of two comparison groups, the statistical significance was calculated using unpaired Student's t tests. For nonnormally distributed variables, Mann-Whitney U tests were utilized to estimate the statistical significance between two groups. When comparing more than two groups, one-way ANOVA tests were used as parametric methods, while Kruskal-Wallis tests were applied as nonparametric methods. Linear relationship determination is often analyzed by Pearson's or Spearman's correlation analysis between two groups. R software and SPSS software were applied in all statistical analyses, and the two software programs were version 4.0.3 and version 26.0, respectively. For experiments, three or more repetitions were usually performed to solidify the results and conclusion, and all data are shown as the mean ± standard deviation (SD). Statistical significance can be found in each figure legend.

## Results

### Immune and stromal score calculation and clinical characteristics of CRC patients

For the purpose of immune and stromal score calculation 22, a total of 480 TCGA-COAD patients were included in our study. The range of immune scores ranged from -700.98670 to 5995.663, while the stromal scores ranged from -2229.1407 to 5043.415. A total of 1932 immune score-related DEGs (differentially expressed genes) and 2654 stromal score-related DEGs are presented on the volcano plot (Supplementary Figure S1A-B). Thirteen coexpressed gene modules were recognized based on WGCNA (weighted correlation network analysis) (Supplementary Figure S1C-D). The green-yellow and yellow modules showed strong relevance to stromal scores, while the magenta, green-yellow and yellow modules were strongly related to immune scores (Supplementary Figure S1D). The intersection of the DEGs related to immunity and stroma is shown in the Venn diagram (Supplementary Figure S1E, F). According to the LASSO-Cox regression (least absolute shrinkage and selection operator), 22 immune-related genes and 15 stromal-related genes were selected as the hub genes. These genes were rigorously analyzed by stepwise Cox regression based on the Akaike information criterion (AIC). And BST2 and CAV1 (caveolin-1) were eventually selected for further study (Supplementary Figure S1G, H). In addition, a nomogram (based on risk score, TNM stage, age and sex) was established (Supplementary Figure S2A-B). The C-index of the nomogram was 0.763 (95% CI = 0.71204-0.814, *p* < 0.001). The survival probability calibration curves demonstrated excellent discrimination and accuracy of this nomogram to predict the probability of 1-year, 3-year and 5-year survival of patients (Supplementary Figure S2C).

According to recent reports, both CAV1 and BST2 are highly associated with the cell proliferation and metastasis of different cancers 24-26. In particular, the metabolism of CAV1 has been well-depicted in the TME of CRC 27. Although the prognostic role of BST2 in CRC has been reported in several studies 20, 21, 28, its potential function in CRC still needs further investigation.

### BST2 is a potential biomarker for predicting immunotherapy of colon cancer

**Figure 1:**
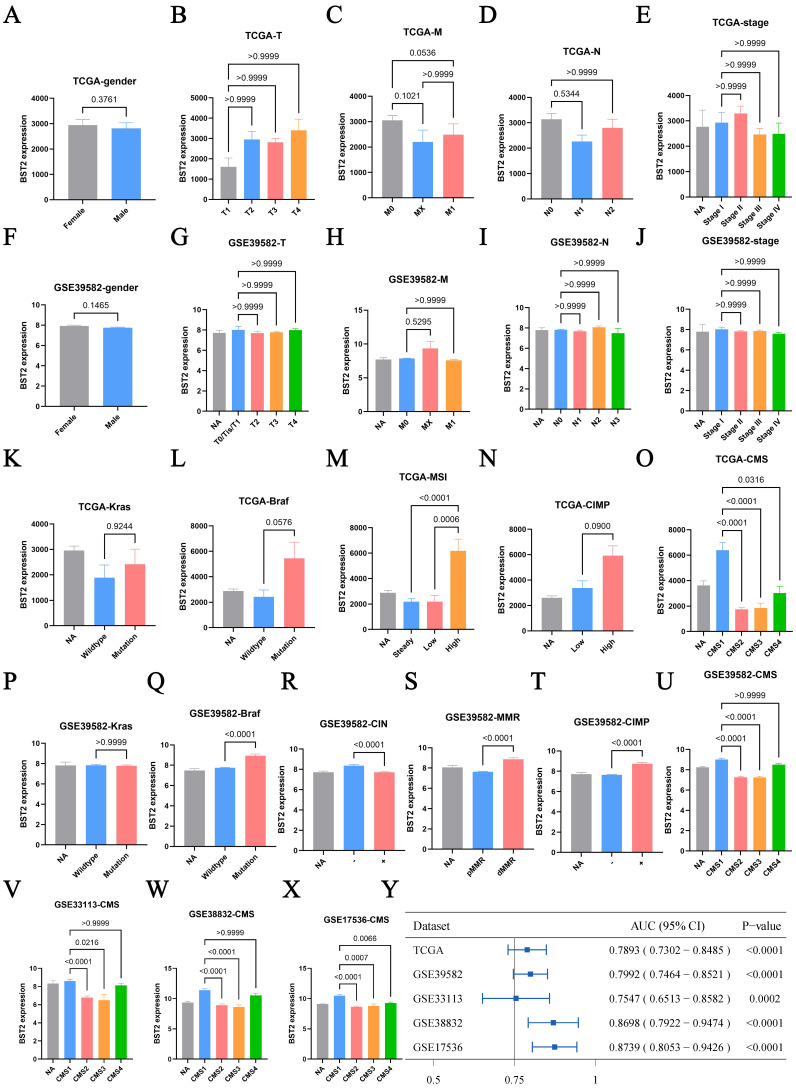
** Clinical characteristic analysis of BST2 in CRC. A-X** Difference analysis of the distribution of BST2 expression in gender, TNM stage, Kras mutation, Braf mutation, MSS/MSI-L/MSI-H, dMMR/pMMR, CIMP and CMS in TCGA and GSE39582. **Y** Forest plot shows the ROC analysis of BST2 in predicting the CMS1 status in TCGA-COAD cohort, GSE39582, GSE33113, GSE38832 and GSE17536. (95% CI: 95% confidence interval). **Abbreviations:** MSS, microsatellite stability; MSI-L, low levels of microsatellite instability; MSI-H, high levels of microsatellite instability; dMMR, mismatch-repair-proficient; pMMR, mismatch-repair-proficient; CIMP, CpG island methylator phenotype; CMS, consensus molecular subtypes.

First, the distribution of BST2 in CRC clinical features was assessed. BST2 expression was demonstrated to have no significant correlation with sex or TNM stage of colon cancer (Figure 1A-J), which directed us to turn to other CRC phenotypes relative to the immune-related genes. In our results, high BST2 expression was validated to be correlated with MSI-H and dMMR in the TCGA-COAD cohort and GSE39582, respectively (Figure 1M, 1S).

Interestingly, our results showed that increased BST2 expression was associated with BRAF-mutation, high CIMP (CpG island methylator phenotype) and low CIN status (chromosomal instability) (Figure 1L, 1N, 1Q-R, 1T). Although high expression of BST2 was a significant difference in both the CMS1 and CMS4 subtypes (consensus molecular subtypes) as compared with the CMS2 and CMS3 subtypes (Figure 1O, 1U-Y), the phenotypic features of BST2 were more compatible with CMS1. BST2 played a relatively excellent role in distinguishing between CMS1 and CMS2-4, with an AUC of over 0.75 in various datasets (TCGA-COAD cohort, GSE39582, GSE33113, GSE38832 and GSE17536).

**Figure 2:**
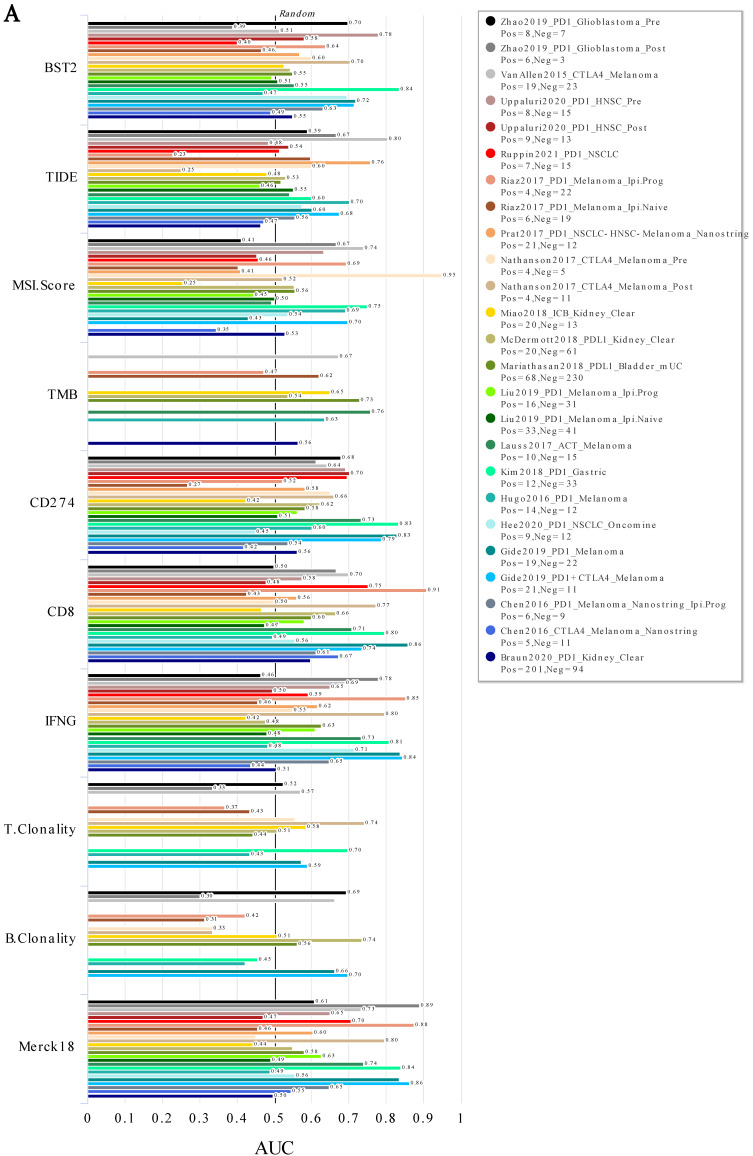
** ICB response in various cancers. A** ROC analysis of BST2, TIDE, MSI. Score, TMB, CD274, CD8, IFNG, T. Clonality, B. Clonality and Merck18 in predicting the ICB response in various cancers. **Abbreviations:** TIDE 37, Tumor Immune Dysfunction and Exclusion; TMB, Tumor Mutational Burden; IFNG, Interferon Gamma; ICB, Immune Checkpoint Blockade

In addition, the analysis of immunotherapy indicated that BST2 showed an effective ability in predicting immunotherapy, especially in gastric cancer, which is relatively closer to CRC (Figure 2A). More interestingly, it played a better role in predicting pretherapy (Figure S5A-B) than posttherapy (Figure 5SC-D). Furthermore, WB, qRT-PCR and flow cytometry assays demonstrated that silencing BST2 in CRC cells could obviously inhibit the expression of PD-L1 (Figure S5E-I).

### BST2 was associated with an immune response

**Figure 3:**
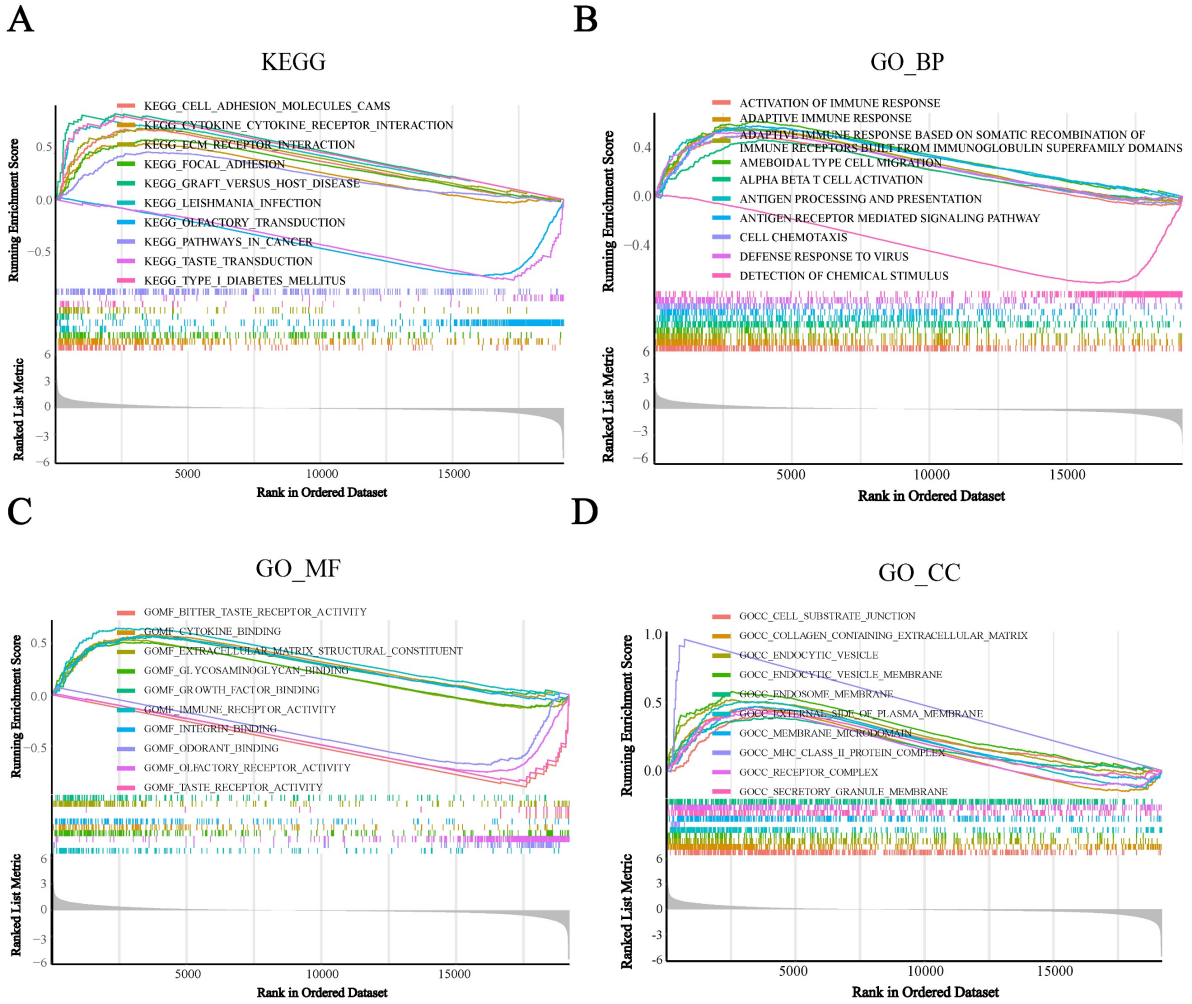
** Gene set enrichment analysis (GSEA) analysis** was performed in the high-BST2 and low-BST2 groups of TCGA. The signaling pathways of positive enrichment scores were enriched in the high-BST2 group, vice versa. **A** KEGG analysis of the TCGA-COAD datasets. **B** GO biological process (GO_BP) analysis in the TCGA-COAD datasets. **C** GO molecular function (GO_MF) analysis in the TCGA-COAD datasets. **D** GO cellular component (GO_CC) analysis in the TCGA-COAD datasets. **Abbreviations:** COAD, colorectal cancer

To assess the potential biological functions of BST2, we utilized gene set enrichment analysis (GSEA) to analyze the TCGA-COAD cohorts. It showed that immune-related pathways were mainly enriched. For the pathways annotated by KEGG analysis, some signaling pathways, such as cytokine receptor interaction, cell adhesion molecules CAMs, ECM receptor interaction and focal adhesion, were significantly enriched in high-BST2 colon cancer (Figure 3A). For the pathways annotated by GO biological process analysis, some of the upgraded pathways enriched in BST2-high colon cancer were as follows: immune response pathways, such as activation of immune response, adaptive immune response, adaptive immune response based on somatic recombination of immune receptors built from the immunoglobulin superfamily domain and alpha-beta T-cell activation (Figure 3B). GO molecular function analysis demonstrated that immune-related pathways such as cytokine binding and immune receptor activity were correlated with BST2-high colon cancers (Figure 3C). Subsequently, other immune-associated pathways, such as the MHC class II protein complex and receptor complex, were also significantly upregulated in the BST2-high cohorts, as shown in the GO cellular component analysis (Figure 3D). According to the GSEA results, the overexpression of BST2 was significantly correlated with the immune response in the tumor microenvironment in colon cancer.

**Figure 4:**
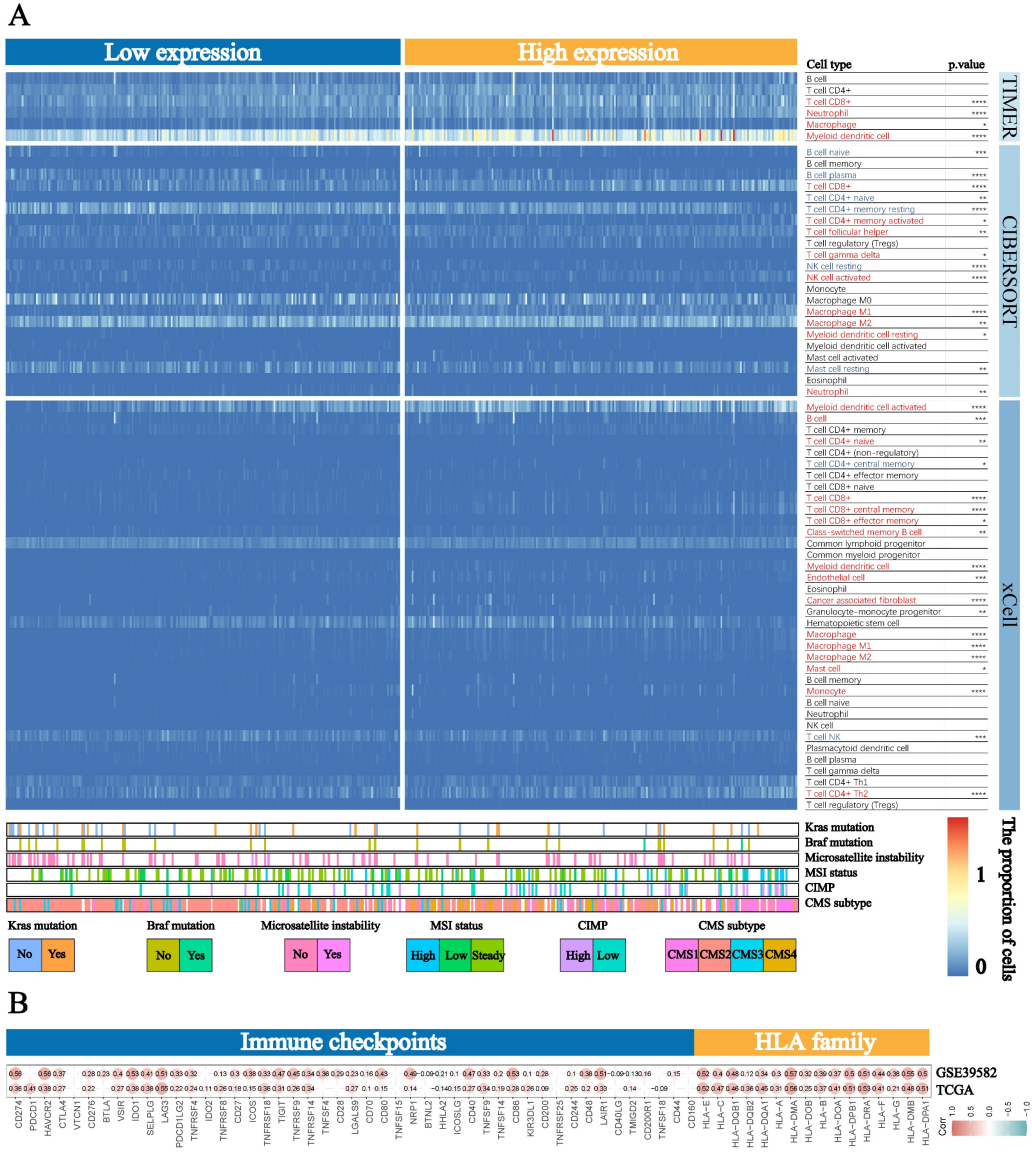
** Landscape of immune cells in the low-BST2 and high-BST2 groups. A** The heatmap shows the normalized proportion of immune cells. Different colors represent different density of immune cells. **B** The correlation analysis between BST2 and immune checkpoints and HLA family genes.

Based on the strong correlation between BST2 and the immune response, the distribution of infiltrating immune cells in different BST2 expression groups inferred by TIMER, CIBERSORT and xCell was also explored. A number of immune cells were either positively or negatively correlated with the expression of BST2 (Figure 4A). Between the high- and low-BST2 groups, we investigated human leukocyte antigen (HLA) family genes and 47 immune checkpoints. Eventually, 16 of the former and 32 of the latter were demonstrated to be tremendously relevant to the group with high BST2 expression and were therefore evaluated and validated both in TCGA-COAD datasets and GSE39582, including HLA-DRA, CD274, CTLA4, IDO1 and LAG3 (Figure 4B). The analysis of other cancers also demonstrated that BST2 was strongly associated with immune cell infiltration and immune response-related molecules (Supplementary Figure S3A-D).

### BST2 was associated with immune cell infiltration in CRC

**Figure 5:**
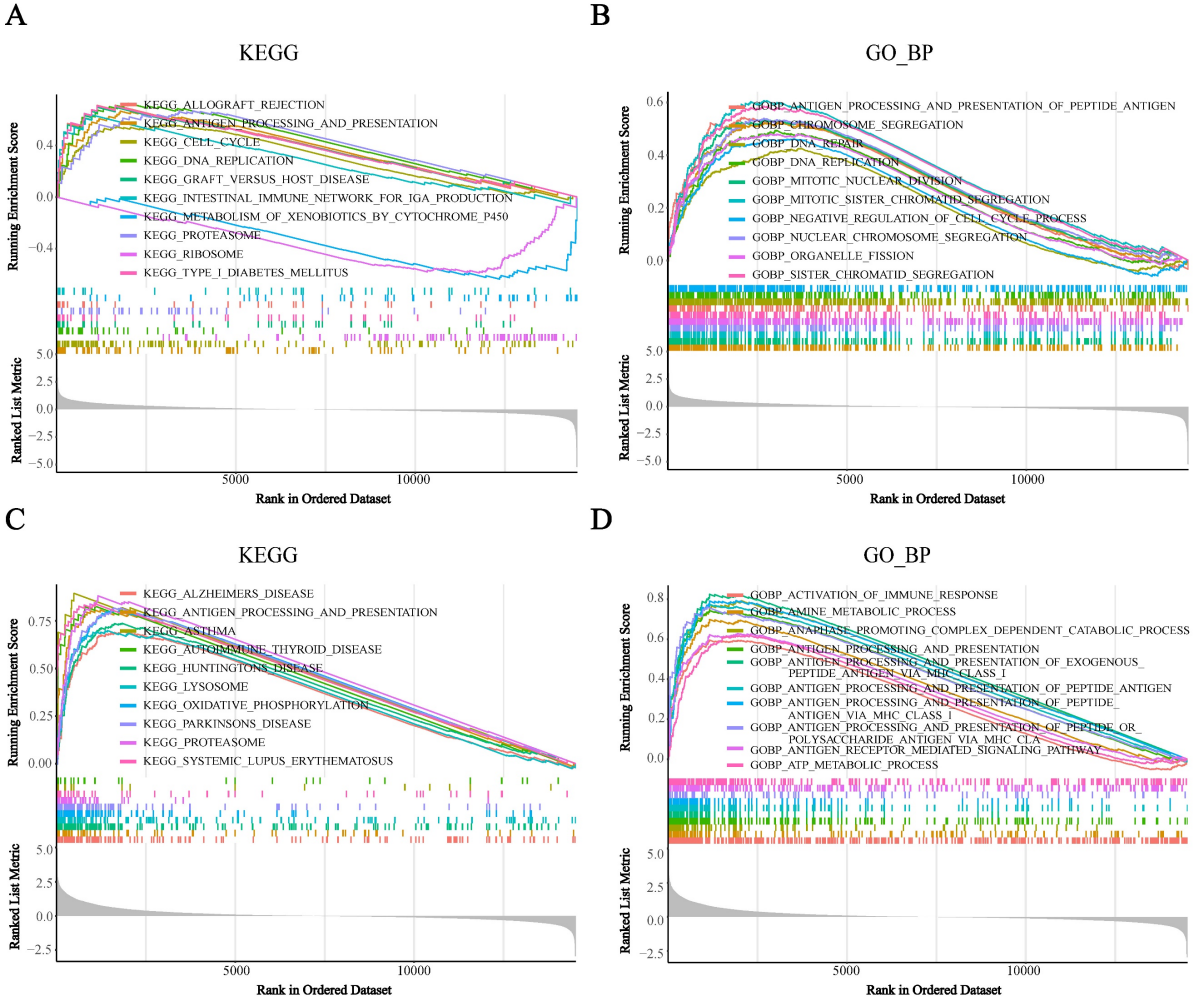
** Gene set enrichment analysis (GSEA) analysis was performed in the high-BST2 and low-BST2 groups in a single cell RNA-seq dataset (GSE146771). A** KEGG analysis of tumor cells. B GO biological process (GO_BP) analysis of tumor cells. C KEGG analysis of monocyte/macrophage. D GO biological process (GO_BP) analysis of monocyte/macrophage.

To further reveal how BST2 was distributed in different types of infiltrating immune cells, three single-cell RNA-seq datasets (GSE146771, GSE146771 and GSE139555) of CRC were analyzed. BST2 was most frequently observed in immune cells and had a relatively high expression in macrophages (Supplementary Figure S4A-C). In addition, the KEGG and GO analyses of the single- cell RNA-seq dataset (GSE146771) suggested that increased expression of BST2 was strongly connected to the cell growth pathways such as the cell cycle and DNA replication pathways in CRC (Figure 5A-B), and immune response-related pathways such as the antigen processing and presentation pathway and activation of the immune response pathway were enriched in macrophages (Figure 5C-D).

### BST2 played a crucial role in the progression of CRC and macrophage M2 polarization

**Figure 6:**
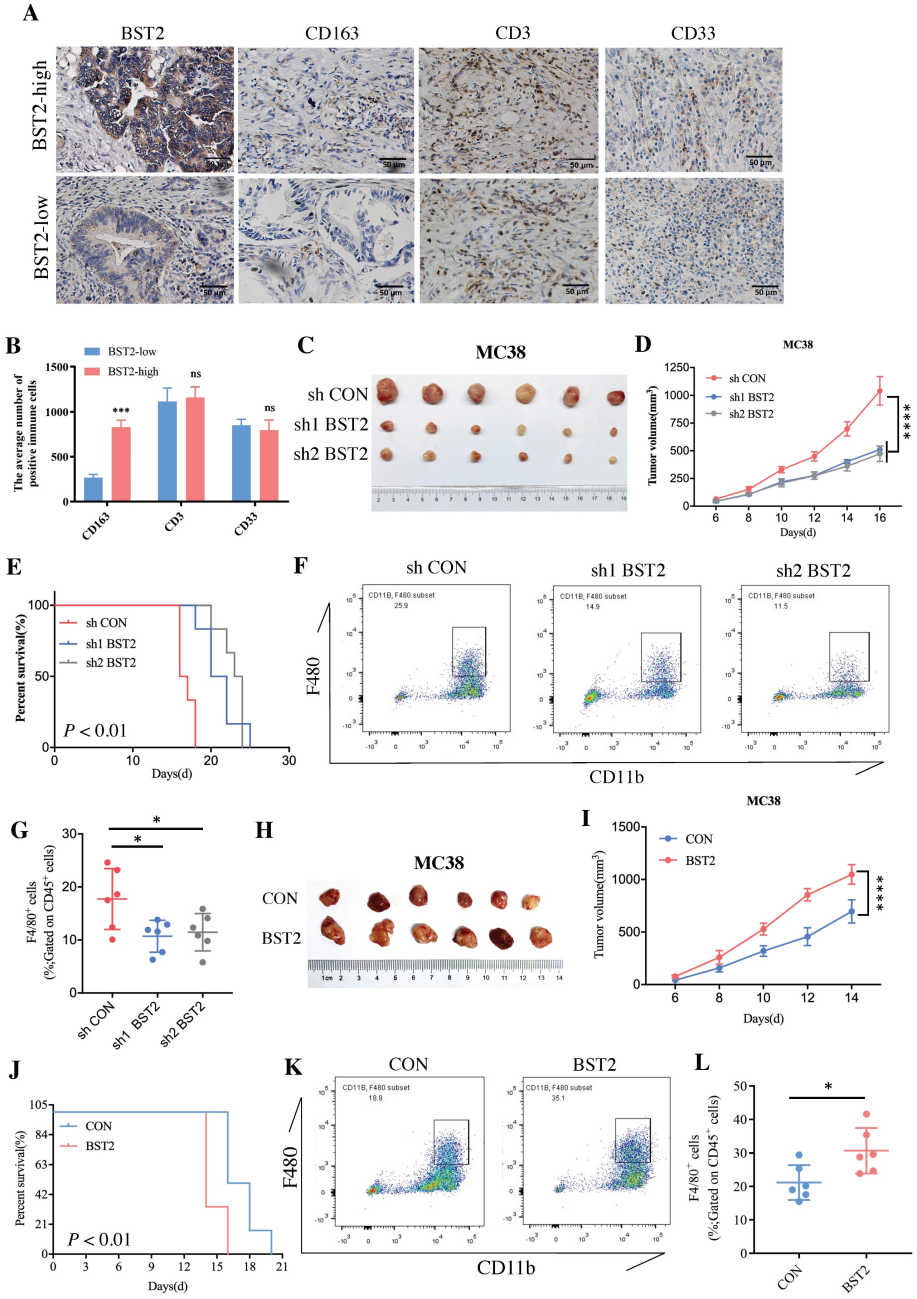
**BST2 promotes CRC progression and TAMs infiltration *in vivo*. A** IHC staining of BST2, TAMs (for the M2 macrophage marker CD163), T cells (CD3) and MDSCs (CD33) in high- and low-BST2 groups. Scale bar=50 μm. **B** The quantitative analysis of IHC staining. **C-E** The tumor size and survival rate of subcutaneous tumor models in the control and BST2 knockdown groups. **F-G** The flow cytometry analysis for the comparison of infiltrated TAMs isolated from subcutaneous tumors between control and BST2 knockdown groups. **H-J** The size of subcutaneous tumors and survival rate of mouse models in the control and BST2 overexpressed groups. **K-L** The flow cytometry analysis for the comparison of infiltrated TAMs isolated from subcutaneous tumors between control and BST2 overexpressed groups. Error bars, SD, *P < 0.05; ***P < 0.001 ****P < 0.0001; ns, not significant.

Although a large number of immune cells that infiltrated the tumor microenvironment were associated with the high expression of BST2, determining the main type of immune cell that contributed to the interaction with cancer cells would be interesting. The representative image of immunohistochemistry (IHC) results of 40 cases of CRC clinical specimens showed that high expression of BST2 expression tended to be associated with the infiltration of more M2 macrophages (CD163 positive) instead of lymphocytes (CD3 positive) and myeloid-derived suppressor cells (MDSCs) (CD33 positive) (Figure 6A-B). We constructed an MC38 cell line stably expressing and knocking down BST2 respectively (Figure S5F-G). *In vivo* experiments demonstrated that knocking down BST2 expression could significantly inhibit the proliferation of CRC while overexpression of BST2 enhanced tumor progression (Figure 6C-E, 6H-J). In addition, our results showed a remarkable decrease in macrophages isolated from BST2 downregulated subcutaneous tumors compared with the control group (Figure 6F-G), while BST2-overexpressing subcutaneous tumors had an increase in macrophages (Figure 6K-L).

**Figure 7:**
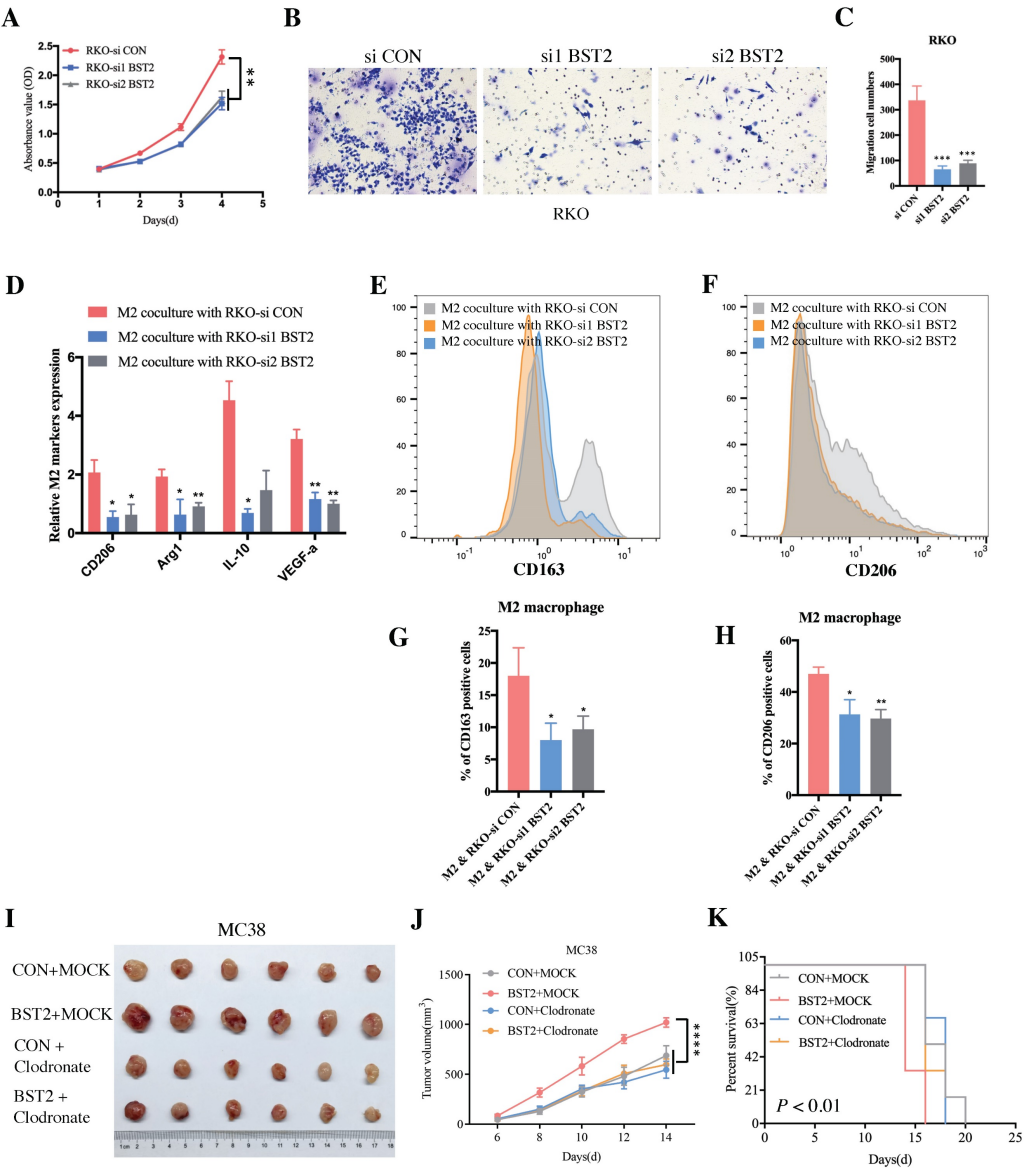
** BST2 promotes CRC progression and TAMs polarization *in vitro*. A** BST2-knockdown RKO cells were subjected to CCK-8 proliferation assay. **B-C** BST2-knockdown RKO cells were subjected to transwell migration assay (left) (scale bar = 50um) and statistical analysis was performed (right). **D-H** qRT-PCR analysis (D) and flow cytometry analysis (E-F) were applied to demonstrate the M2 polarization when TAMs were cocultured with BST2-knockdown RKO cells and G-H were statistical analysis of E-F, respectively. **I-K** The size of subcutaneous tumors (I-J) and survival rate (K) of mouse models in the MC38 + control, MC38 + BST2 knockdown, MC38 + clodronate and BST2 overexpression + clodronate groups. *P < 0.05; **P < 0.01; ***P < 0.001 ****P < 0.0001.

*In vitro* experiments further demonstrated that downregulated BST2 expression could significantly inhibit tumor proliferation (Figure 7A) and migration (Figure 7B-C). There was a marked cell cycle arrest in the S phase after the knockdown of BST2 in CRC cells, which further explained the proliferation inhibition of CRC cells after BST2 knockdown (Figure S5L-M).

Moreover, BST2 not only promoted the proliferative and migrative ability of CRC cells but also facilitated M2 polarization (CD163 positive and CD206 positive) (Figure 7D-H) instead of influencing M1 polarization (Figure S5N). Importantly, with the application of clodronate to deplete macrophages *in vivo*, it could be concluded that tumor-associated macrophages served as a critical characteristic in the progression of CRC (Figure 7I-K).

## Discussion

The significance of the TME in CRC promoted us to determine the pivotal genes in the TME. In this study, according to the ESTIMATE algorithm and subsequent bioinformatics analysis, BST2 and CAV1 were finally identified as candidate genes and constructed into the risk score. With a combination of risk score and clinical features, a nomogram was built, and it showed a satisfactory prognostic value. When faced with BST2 and CAV1, we focused on exploring BST2 because of the mysterious role of BST2 in CRC. Previous studies revealed that BST2 could be a prognostic marker in CRC 20, 21. However, whether BST2 could serve as a potential predictive biomarker for immunotherapy remained unknown. Interestingly, high expression of BST2 was strongly associated with the dMMR/MSI-H and CMS1 phenotypes, which suggested that CRC patients with high BST2 expression may also be suitable for immunotherapy. Subsequent analysis demonstrated the powerful ability of BST2 in predicting immune checkpoint blockade therapy. CRC is one of the most common cancers worldwide and often has a high death rate. However, despite the improvement of therapeutic strategies, they still show poor therapeutic efficacy for advanced CRC patients, with a median 5-year survival rate of only 12.5% 1. In 2017, immune checkpoint blockade therapy received regulatory authorization for CRC patients in dMMR/MSI-H status. In contrast, immunotherapy is usually ineffective for mismatch-repair-proficient (pMMR) and microsatellite-stable (MSS) tumors or tumors with low levels of microsatellite instability (MSI-L) are usually ineffective 29, 30. However, only 15% of CRC patients are in dMMR/MSI-H status, and there are fewer dMMR/MSI-H tumors in stage IV, which account for approximately 2% to 4% of all mCRCs 31, 32.

Consensus molecular subtypes (CMSs) were set up to describe the features of CRC in a previous study; and according to their description, CMS1 is characterized as an emerging feature of MSI CRC with a diffuse immune infiltrate and strong activation of the immune evasion pathway. In addition, CMS1 has a CpG island methylator phenotype (CIMP)-higher status, more frequent occurrence of BRAF mutations and lower chromosomal instability (CIN) than CMS2-CMS4 33. High expression of BST2 was not only associated with dMMR/MSI-H but also corelated with CMS1. Our results of immunotherapeutic analysis indicated that BST2 could be a critical biomarker in predicting immune checkpoint blockade therapy before patients began to take therapy in CRC. CMS1 consists of the majority of MSI tumors and a small number of MSS tumors 33. According to our results, BST2 might play a critical role in enrolling a small population that is MSS but BST2 positive in immune checkpoint blockade therapy.

The GSEA of the CRC sing-cell RNA-seq dataset (GSE146771) found that cell growth pathways, such as the cell cycle and DNA replication pathways, were enriched. Consistently, subsequent *in vitro* experiments demonstrated that inhibiting BST2 could obviously restrain the cell cycle in the S phase. In addition, repressing the expression of BST2 noticeably decreased the proliferative and migrative ability of CRC cells, which further confirmed the conclusions of previous studies 20, 21. Dysregulation of BST2 expression has also been studied in several human cancers. All studies have revealed the oncogenic role of BST2. BST2 has the potential to enhance the ability of cell proliferation, apoptosis and motility by activating the NF-κB signaling pathway in gastric cancer 34. In oral squamous cell carcinoma, high BST2 expression could induce gefitinib resistance by regulating the EGFR pathway 35. Similar explorations have also been investigated in myeloma 15, breast cancer 16, lung cancer 17 and kidney cancer 18.

GSEA of TCGA-COAD cohorts showed that increasing BST2 in CRC had strong relevance to immune-associated pathways. The exploration of the distribution of immune cell infiltration provided an aspect for us to investigate the potential function of TAMs affected by CRC. In general, TAMs were definitely the major type of immune cells that infiltrated in the TME. TAMs have been widely accepted to form a favorable condition for tumor progression and immune suppression in the TME. Therefore, it would be critical to suppress TAMs-mediated tumor progression if the crucial factors in M2 macrophage polarization were understood 36. We found that the number of infiltrated CD163^+^ macrophages was significantly associated with the high expression of BST2 in CRC tissue samples. It could be concluded from* in vivo* and* in vitro* experiments that upregulation of BST2 in CRC increased the infiltration ratio of TAMs by recruiting them and educating them to the M2 phenotype, which helped form an immune-suppressive TME. Furthermore,* in vivo* experiments demonstrated that the depletion of macrophages could neutralize the effects of BST2 overexpression in CRC. A previous study demonstrated that BST2 regulated by FGD5-AS1 could promote M2 macrophage polarization and inhibit M1 macrophage polarization in cervical cancer 24. Both results identified the oncogenic contribution of BST2 in shaping a suppressive microenvironment.

## Supplementary Material

Supplementary figures.

## Abbreviations

TME: tumor microenvironment
CRC: colorectal cancer
TAMs: tumor-associated macrophages
BST2: Bone Marrow Stromal Cell Antigen 2
AIC: Akaike information criterion
CAV1: Caveolin 1
dMMR: mismatch-repair-deficient
MSI-H: high levels of microsatellite instability
mCRC: metastatic CRC
GSEA: gene set enrichment analysis
HLA: human leukocyte antigen
IHC: immunohistochemistry
MDSCs: myeloid-derived suppressor cells
pMMR: mismatch-repair-proficient
MSS: microsatellite-stable
MSI-L: low levels of microsatellite instability
CMS: consensus molecular subtype
CIMP: CpG island methylator phenotype
CIN: chromosomal instability
DEG: differentially expressed gene
FDR: false discovery rate
FC: Fold Change
WGCNA: weighted correlation network analysis
C‐index: concordance index
GO: gene ontology
KEGG: Kyoto encyclopedia of genes and genomes
qRT-PCR: quantitative real-time PCR
DAB: 3,3-diaminobenzidine
CCK-8: cell counting kit-8
PI: propidium iodide
SD: standard deviation
