# Supplementary material for: BST2 induced macrophage M2 polarization to promote the progression of colorectal cancer
Source: Int J Biol Sci. 2023 Jan 1;19(1):331–45. doi: 10.7150/ijbs.72538 (PMC9760448; doi:10.7150/ijbs.72538)
Supplement: Supplementary file 1 — Supplementary figures. [file ijbsv19p0331s1.pdf]

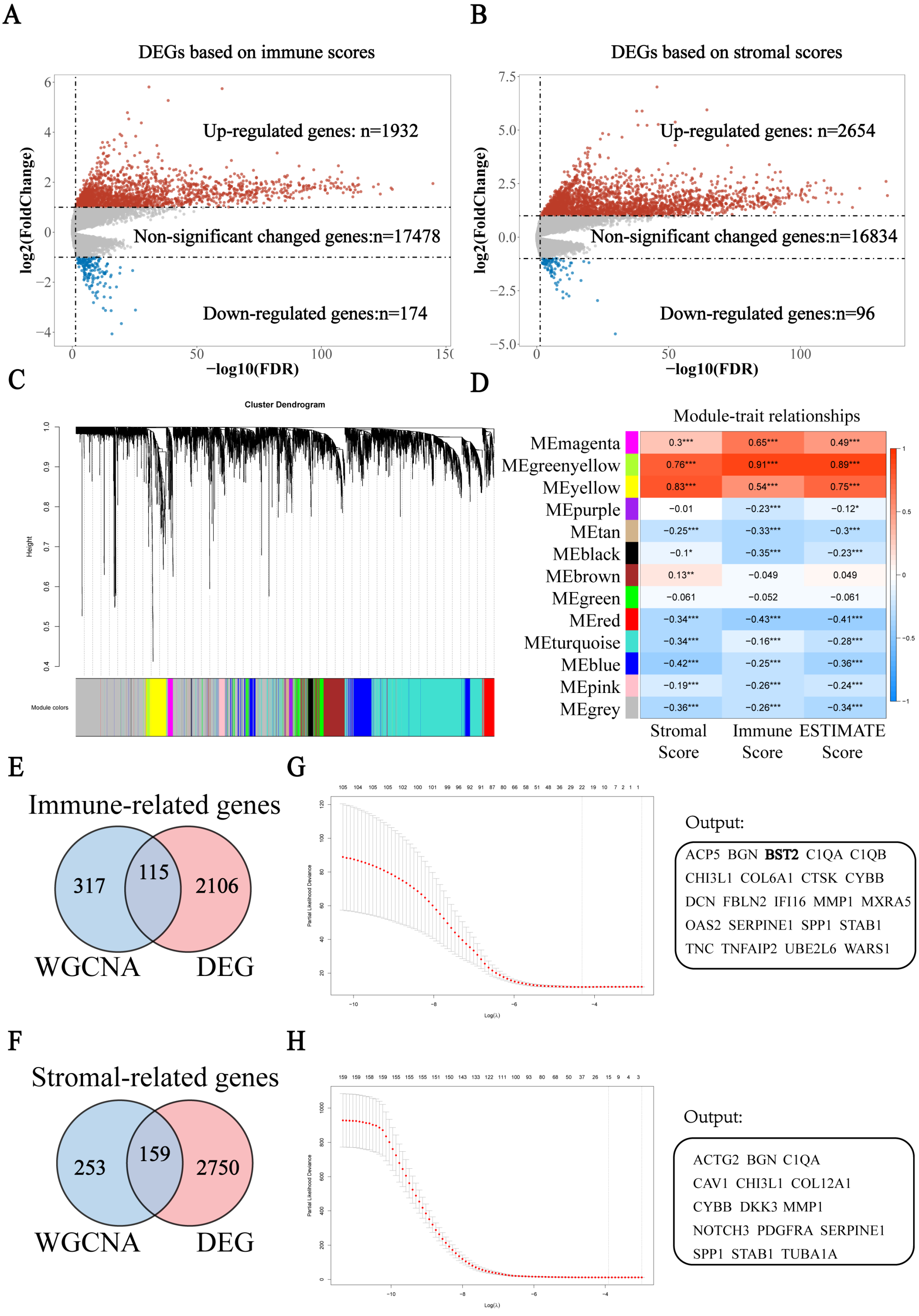
A
DEGs based on immune scores
Up-regulated genes: n=1932
Non-significant changed genes:n=17478
Down-regulated genes:n=174
log2(FoldChange)
−log10(FDR)
B
DEGs based on stromal scores
Up-regulated genes: n=2654
Non-significant changed genes:n=16834
Down-regulated genes:n=96
log2(FoldChange)
−log10(FDR)
C
Cluster Dendrogram
Height
Module colors
D
Module-trait relationships
MEmagenta 0.3*** 0.65*** 0.49***
MEgreenyellow 0.76*** 0.91*** 0.89***
MEyellow 0.83*** 0.54*** 0.75***
MEpurple −0.01 −0.23*** −0.12*
MEtan −0.25*** −0.33*** −0.3***
MEblack −0.1* −0.35*** −0.23***
MEbrown 0.13** −0.049 0.049
MEgreen −0.061 −0.052 −0.061
MEred −0.34*** −0.43*** −0.41***
MEturquoise −0.34*** −0.16*** −0.28***
MEblue −0.42*** −0.25*** −0.36***
MEpink −0.19*** −0.26*** −0.24***
MEgrey −0.36*** −0.26*** −0.34***
Stromal Score
Immune Score
ESTIMATE Score
E
Immune-related genes
317
115
2106
WGCNA
DEG
G
Partial Likelihood Deviance
Log(λ)
Output:
ACP5 BGN BST2 C1QA C1QB
CHI3L1 COL6A1 CTSK CYBB
DCN FBLN2 IFI16 MMP1 MXRA5
OAS2 SERPINE1 SPP1 STAB1
TNC TNFAIP2 UBE2L6 WARS1
F
Stromal-related genes
253
159
2750
WGCNA
DEG
H
Partial Likelihood Deviance
Log(λ)
Output:
ACTG2 BGN C1QA
CAV1 CHI3L1 COL12A1
CYBB DKK3 MMP1
NOTCH3 PDGFRA SERPINE1
SPP1 STAB1 TUBA1A

Figure S1 **Screening for targeted genes. A** DEG analysis between low and high immune scores. **B** DEG analysis between low and high stromal scores. **C** WGCNA analysis for identifying gene modules. **D** Correlation between immune, stromal and ESTIMATE scores and gene modules. *P < 0.05; **P < 0.01; ***P < 0.001. **E** Venn plot displays the number of immune-related genes between DEG analysis and WGCNA. **F** Venn plot displays the number of stromal-related genes between DEG analysis and WGCNA. **G** The immune-related genes were further inputted into LASSO analysis. **H** The intersection stromal-related genes were further inputted into LASSO analysis. The Y-axis shows LASSO coefficients and the X-axis is −log (lambda). And the output genes were in the right rectangle boxes.

Abbreviations: DEG, differentially expressed gene); WGCNA, weighted correlation network analysis ; LASSO, Least absolute shrinkage and selection operator

A

Factor	Univariate Cox analysis		Multivariate Cox analysis	
	HR(95% CI)	p value	HR(95% CI)	p value
riskscore	2.545 (1.584-4.090)	<0.001	2.080 (1.313-3.295)	0.002
M	4.052 (2.593-6.333)	<0.002	1.946 (0.881-4.299)	0.1
N	1.961 (1.560-2.464)	<0.003	1.340 (0.899-2.000)	0.151
T	2.411 (1.648-3.527)	<0.004	1.789 (1.120-2.859)	0.015
Stage	2.046 (1.628-2.571)	<0.005	1.266 (0.711-2.252)	0.422
Age	1.028 (1.010-1.046)	0.002	1.036 (1.018-1.054)	<0.001

B

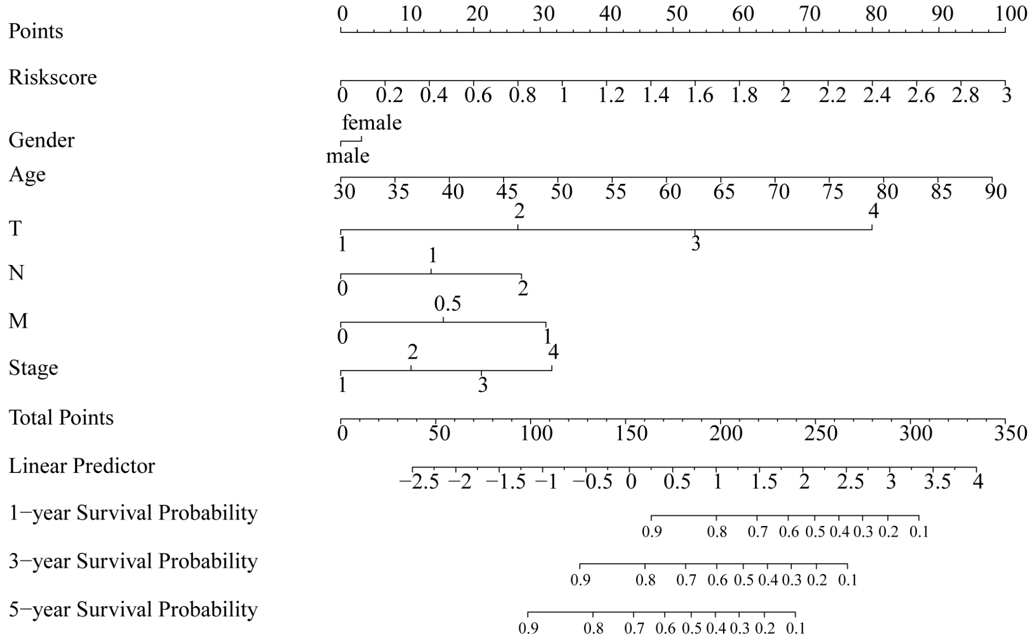

C

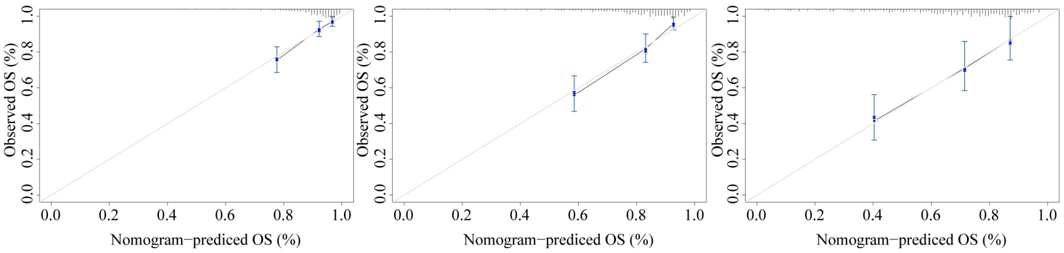

Figure S2 **Prognostic analysis of BST2 in CRC.** **A** Univariate and multivariate Cox regression analysis of riskscore, TNM stage, age and gender. **B** Prognostic nomograms based on risk score, TNM stage, age and gender. **C** Calibration analysis of the nomogram for predicting 1-year, 3-year and 5-year overall survival, respectively.

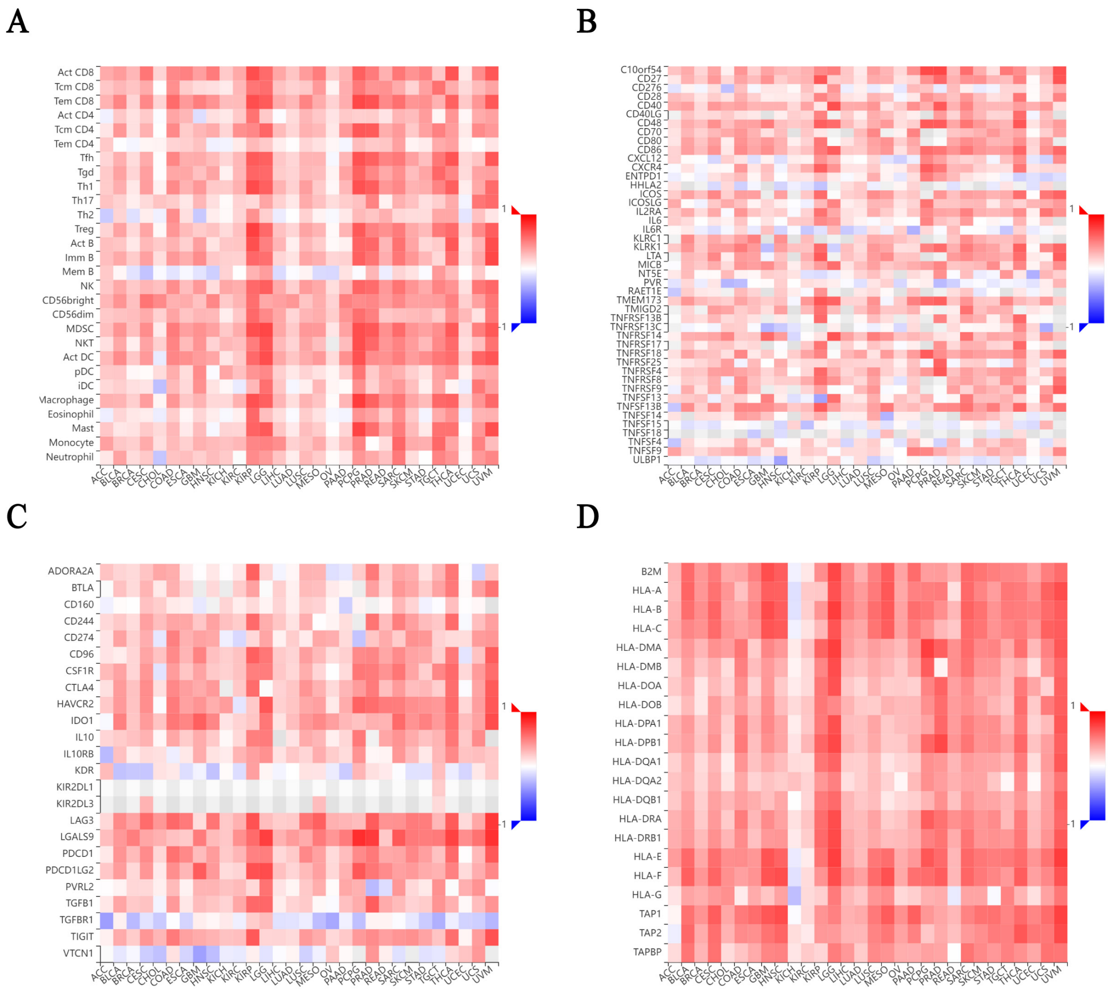
A
Act CD8
Tcm CD8
Tem CD8
Act CD4
Tcm CD4
Tem CD4
Tfh
Tgd
Th1
Th17
Th2
Treg
Act B
Imm B
Mem B
NK
CD56bright
CD56dim
MDSC
NKT
Act DC
pDC
iDC
Macrophage
Eosinophil
Mast
Monocyte
Neutrophil
ACC BLCA BRCA CESC CHOL COAD ESCA GBM HNSC KICH KIRC KIRP LGG LIHC LUAD LUSC MESO OV PAAD PCPG PRAD READ SARC SKCM STAD TGCT THCA UCEC UCS UVM
1
-1
B
C10orf54
CD27
CD276
CD28
CD40
CD40LG
CD48
CD70
CD80
CD86
CXCL12
CXCR4
ENTPD1
HHLA2
ICOS
ICOSLG
IL2RA
IL6
IL6R
KLRC1
KLRK1
LTA
MICB
NT5E
PVR
RAET1E
TMEM173
TMIGD2
TNFRSF13B
TNFRSF13C
TNFRSF14
TNFRSF17
TNFRSF18
TNFRSF25
TNFRSF4
TNFRSF8
TNFRSF9
TNFSF13
TNFSF13B
TNFSF14
TNFSF15
TNFSF18
TNFSF4
TNFSF9
ULBP1
ACC BLCA BRCA CESC CHOL COAD ESCA GBM HNSC KICH KIRC KIRP LGG LIHC LUAD LUSC MESO OV PAAD PCPG PRAD READ SARC SKCM STAD TGCT THCA UCEC UCS UVM
1
-1
C
ADORA2A
BTLA
CD160
CD244
CD274
CD96
CSF1R
CTLA4
HAVCR2
IDO1
IL10
IL10RB
KDR
KIR2DL1
KIR2DL3
LAG3
LGALS9
PDCD1
PDCD1LG2
PVRL2
TGFB1
TGFBR1
TIGIT
VTCN1
ACC BLCA BRCA CESC CHOL COAD ESCA GBM HNSC KICH KIRC KIRP LGG LIHC LUAD LUSC MESO OV PAAD PCPG PRAD READ SARC SKCM STAD TGCT THCA UCEC UCS UVM
1
-1
D
B2M
HLA-A
HLA-B
HLA-C
HLA-DMA
HLA-DMB
HLA-DOA
HLA-DOB
HLA-DPA1
HLA-DPB1
HLA-DQA1
HLA-DQA2
HLA-DQB1
HLA-DRA
HLA-DRB1
HLA-E
HLA-F
HLA-G
TAP1
TAP2
TAPBP
ACC BLCA BRCA CESC CHOL COAD ESCA GBM HNSC KICH KIRC KIRP LGG LIHC LUAD LUSC MESO OV PAAD PCPG PRAD READ SARC SKCM STAD TGCT THCA UCEC UCS UVM
1
-1

Figure S3 **The correlation analysis between BST2 and various immune factors. A** The heatmap showed the correlation between BST2 and immune cells in various cancers. **B** The heatmap showed the correlation between BST2 and immune stimulators in various cancers. **C** The heatmap showed the correlation between BST2 and immune inhibitors in various cancers. **D** The heatmap showed the correlation between BST2 and MHC molecules in various cancers. The plot above were downloaded from http://cis.hku.hk/TISIDB/

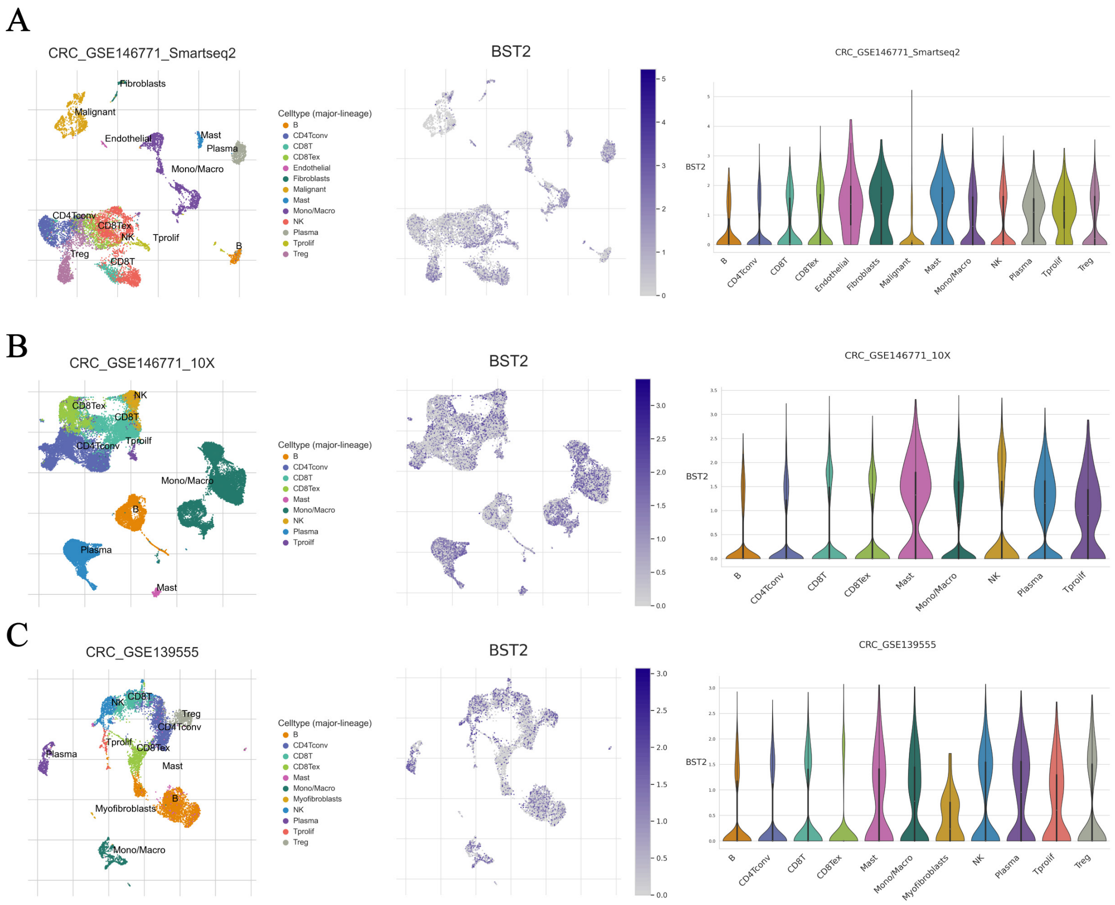
A
CRC_GSE146771_Smartseq2
Fibroblasts
Malignant
Endothelial
Mast
Plasma
Mono/Macro
CD4Tconv
CD8Tex
NK
Tprolif
Treg
CD8T
B
Celltype (major-lineage)
B
CD4Tconv
CD8T
CD8Tex
Endothelial
Fibroblasts
Malignant
Mast
Mono/Macro
NK
Plasma
Tprolif
Treg
BST2
CRC_GSE146771_Smartseq2
BST2
B
CD4Tconv
CD8T
CD8Tex
Endothelial
Fibroblasts
Malignant
Mast
Mono/Macro
NK
Plasma
Tprolif
Treg
B
CRC_GSE146771_10X
NK
CD8Tex
CD8T
CD4Tconv
Tproilf
Mono/Macro
B
Plasma
Mast
Celltype (major-lineage)
B
CD4Tconv
CD8T
CD8Tex
Mast
Mono/Macro
NK
Plasma
Tproilf
BST2
CRC_GSE146771_10X
BST2
B
CD4Tconv
CD8T
CD8Tex
Mast
Mono/Macro
NK
Plasma
Tproilf
C
CRC_GSE139555
NK
CD8T
Treg
CD4Tconv
Plasma
Tprolif
CD8Tex
Mast
Myofibroblasts
B
Mono/Macro
Celltype (major-lineage)
B
CD4Tconv
CD8T
CD8Tex
Mast
Mono/Macro
Myofibroblasts
NK
Plasma
Tprolif
Treg
BST2
CRC_GSE139555
BST2
B
CD4Tconv
CD8T
CD8Tex
Mast
Mono/Macro
Myofibroblasts
NK
Plasma
Tprolif
Treg

Figure S4 **Distribution of BST2 in different types of immune cells analyzed in three single cell RNA-seq datasets.** **A** GSE146771; **B** GSE146771; **C** GSE139555; Different colors represent different kinds of immune cells.

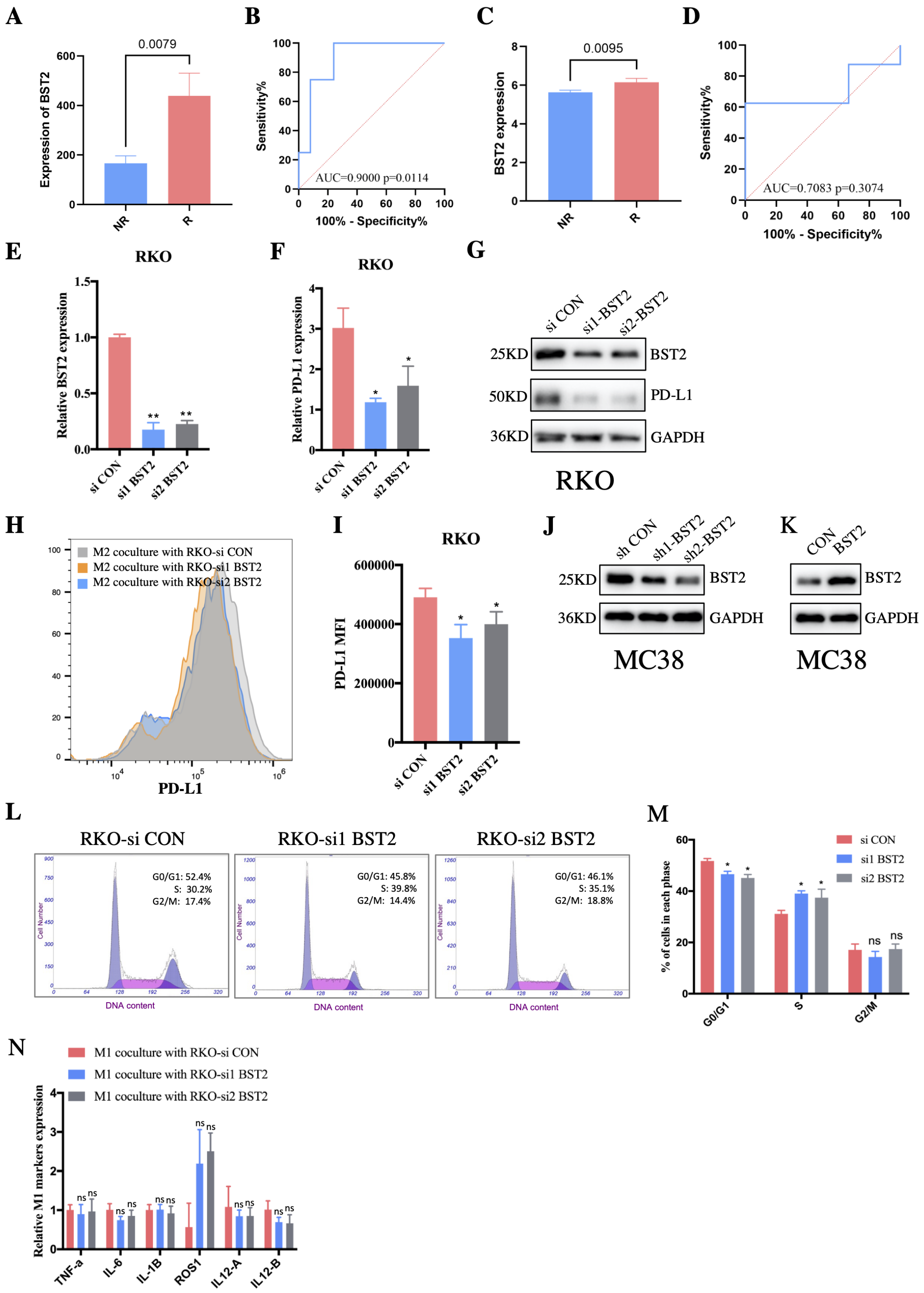

A
0.0079
Expression of BST2
NR
R
B
Sensitivity%
AUC=0.9000 p=0.0114
100% - Specificity%
C
0.0095
BST2 expression
NR
R
D
Sensitivity%
AUC=0.7083 p=0.3074
100% - Specificity%
E
RKO
Relative BST2 expression
si CON
si1 BST2
si2 BST2
F
RKO
Relative PD-L1 expression
si CON
si1 BST2
si2 BST2
G
si CON
si1-BST2
si2-BST2
25KD
BST2
50KD
PD-L1
36KD
GAPDH
RKO
H
M2 coculture with RKO-si CON
M2 coculture with RKO-si1 BST2
M2 coculture with RKO-si2 BST2
PD-L1
I
RKO
PD-L1 MFI
si CON
si1 BST2
si2 BST2
J
sh CON
sh1-BST2
sh2-BST2
25KD
BST2
36KD
GAPDH
MC38
K
CON
BST2
BST2
GAPDH
MC38
L
RKO-si CON
G0/G1: 52.4%
S: 30.2%
G2/M: 17.4%
RKO-si1 BST2
G0/G1: 45.8%
S: 39.8%
G2/M: 14.4%
RKO-si2 BST2
G0/G1: 46.1%
S: 35.1%
G2/M: 18.8%
Cell Number
DNA content
M
si CON
si1 BST2
si2 BST2
% of cells in each phase
G0/G1
S
G2/M
N
M1 coculture with RKO-si CON
M1 coculture with RKO-si1 BST2
M1 coculture with RKO-si2 BST2
Relative M1 markers expression
TNF-a
IL-6
IL-1B
ROS1
IL12-A
IL12-B

Figure S5 **A** Difference analysis of the distribution of BST2 expression between non-responder and responder prior therapy. **B** ROC analysis of BST2 expression prior therapy in predicting the ICB response. **C** Difference analysis of the distribution of BST2 expression between non-responder and responder post therapy. **D** ROC analysis of BST2 expression post therapy in predicting the ICB response. **E-I** qRT-PCR (E-F), Western blot analysis (G) were used to certificate the knockdown of BST2 in RKO, and the downregulation of PD-L1 in RKO. Flow cytometry analysis to further certificate the downregulation of PD-L1 in RKO (H-I). **J** Western blot analysis was used to certificate knockdown of shRNA-BST2 in RKO. **K** Western blot analysis was used to certificate the overexpression of BST2 in RKO. **L** BST2-knockdown RKO cells were applied to cell cycle analysis assays. **M** The percentage of RKO cells in G0/G1, S and G2/M phases. $*P < 0.05$; $**P < 0.01$; ns, not significant. **N** qRT-PCR analysis was applied to demonstrate the M1 polarization when TAMs cocultured with BST2-knockdown RKO cells.
